# Transcriptomics of the *Anthopleura* Sea Anemone Reveals Unique Adaptive Strategies to Shallow‐Water Hydrothermal Vent

**DOI:** 10.1002/ece3.71252

**Published:** 2025-04-10

**Authors:** Mei‐Fang Lin, Li‐Lian Liu, Chen‐Tung Arthur Chen

**Affiliations:** ^1^ Department of Marine Biotechnology and Resources National Sun Yat‐Sen University Kaohsiung Taiwan; ^2^ Doctoral Degree Program in Marine Biotechnology National Sun Yat‐Sen University Kaohsiung Taiwan; ^3^ Frontier Center for Ocean Science and Technology National Sun Yat‐Sen University Kaohsiung Taiwan; ^4^ Department of Oceanography National Sun Yat‐Sen University Kaohsiung Taiwan

**Keywords:** adaptation, *Anthopleura*, detoxification, hydrothermal vent, stress resistance, thermotolerance

## Abstract

The nonsymbiotic sea anemone *Anthopleura nigrescens* dominates the shallow‐water hydrothermal vents off the coast of Kueishan Island, Taiwan. These vents represent some of the world's most extreme environments, with recorded pH values as low as 1.52 and temperatures reaching 121°C. To investigate the adaptations of *A. nigrescens* to these extreme conditions, transcriptomic analyses were conducted to compare populations inhabiting vent and non‐vent areas. To identify shared genetic mechanisms in vent‐dwelling anemones, specific orthologs conserved in vent sea anemones were identified by comparing the genomic data of *Anthopleura* species and other sea anemones. Tank experiments with elevated temperatures were also performed to evaluate the expression profiles of genes associated with heat resistance. The transcriptomic analysis revealed that enriched genes in vent populations are involved in H_2_S homeostasis and stress resistance, suggesting that detoxification and thermal stress resistance are critical adaptive strategies. Two significantly upregulated genes encoding hydroxyacylglutathione hydrolase and thiosulfate sulfurtransferase may play a role in managing sulfur toxicity and maintaining redox balance. The enriched genes and vent‐specific gene expression patterns also suggest that efficient DNA repair mechanisms play a crucial role in the thermal stress resistance of vent populations. Interestingly, some genes associated with circadian rhythms were upregulated in vent populations, suggesting these genes may help vent anemones adapt to the highly dynamic conditions of hydrothermal vents. Furthermore, the expression profiles of stress‐resistance‐related genes reveal that vent anemones have developed unique molecular regulatory mechanisms to cope with elevated temperatures, as observed in the tank experiment. These transcriptomic findings advance our understanding of the life adaptations in shallow‐water hydrothermal vent environments.

## Graphical abstract

The nonsymbiotic sea anemone *Anthopleura nigrescens* thrives in the extreme shallow‐water hydrothermal vents off Kueishan Island, Taiwan, where they represent some of the world's most extreme environments. Transcriptomic analyses revealed that genes involved in H_2_S homeostasis, stress resistance, and DNA repair are enriched in vent populations, highlighting key adaptive strategies. Additionally, upregulated genes related to sulfur detoxification, redox balance, and circadian rhythms suggest unique molecular mechanisms that enable *A. nigrescens* to survive and adapt to the dynamic hydrothermal vent environment.

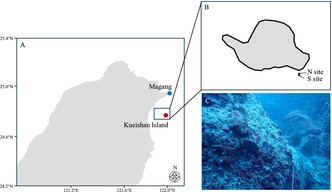

## 1 Introduction

The exploration of organisms' adaptation to extreme environments stands as a captivating frontier in biological research, offering profound insights into the limits of life and the mechanisms driving evolutionary change. Life in hydrothermal vents presents a formidable array of challenges, owing to the extreme conditions of these ecosystems. Located in the dark depths of the ocean floor, deep‐sea hydrothermal vents emit superheated fluids laden with toxic chemicals and heavy metals, creating an environment characterized by high temperatures, pressure, and chemical toxicity. Within this realm, the study of organismal adaptive mechanisms, particularly in the context of hydrothermal vent ecosystems, provides a unique lens through which to understand the intricate interplay between environmental extremes and evolutionary adaptation (Bioy et al. 2022; Tobler et al. 2018; Xu et al. 2022; Zhou et al. 2023). Organisms inhabiting these vents were found to contend with the constant threat of protein denaturation, membrane destabilization, metabolic inhibition, unique gene family expansions, and gene innovations due to the harsh physicochemical conditions (Zhou et al. 2023). The absence of sunlight precludes photosynthesis, necessitating reliance on alternative energy sources such as chemosynthesis, a process that extracts energy from inorganic compounds present in the vent fluids (Fisher 1998; Hand and Somero 1983; Le Layec and Hourdez 2021). Despite these daunting obstacles, life has not only managed to survive but has thrived in the extreme environments of hydrothermal vents, showcasing the remarkable adaptability and resilience of biological systems.

Studies have indicated that shallow‐water vents and deep‐sea vents have very distinct physical and chemical properties, leading to different species communities and ecosystems (Dando 2010; Tarasov 2006; Tarasov et al. 2005). Different from the deep‐sea vents where chemosynthesis generates major energy, energy supplies of shallow‐water vent ecosystems mainly rely on both photosynthesis and chemosynthesis (Dando 2010; Tarasov 2006). Notably, a review of integrated studies on coastal marine ecosystems in the West Pacific suggested that shallow‐water venting areas contain fewer obligate hydrothermal species than deep‐sea vents (Tarasov 2006). Furthermore, studies indicated that species from hydrothermal vents and shallow waters exhibit similar oxygen consumption rates; however, hydrothermal vent species appear to be more sensitive to temperature variations than their shallow‐water counterparts (Le Layec and Hourdez 2021). Knowledge of the life adaptation strategy at the hydrothermal vent is improving, although molecular information is scarcely obtained due to the difficulty of preserving adequate DNA or RNA for genomic assessment. Also, the understanding of substantial molecular machinery associated with life surviving in shallow‐water vents is relatively limited.

Kueishan Island is a volcanic island in northeastern Taiwan with roughly 50 shallow‐water hydrothermal vent discharges of depths ranging from 10 to 80 meters (m). Vents there constantly emit hydrothermal fluids and volcanic gases containing CO_2_, H_2_S, O_2_, and N_2_ (Chen, Wang, et al. 2005; Yang et al. 2005). These vents record some of the most extreme pH values (1.52) and temperatures (121°C) in the world. The vents emit yellow or white plumes (hereafter named YV or WV), with temperature varying from 54°C to 121°C and pH from 1.52–6.32 for YV, while for WV temperature ranges from 30°C to 65°C and pH from 1.84–6.96 (Chen, Zeng, et al. 2005; Chen et al. 2016; Hung et al. 2019; Lebrato et al. 2019; Mei et al. 2022; Yang et al. 2012). Benthic organisms found in the shallow‐water hydrothermal vents off Kueishan Island include filamentous bacteria, red algae, stony coral (*Tubastraea* spp. Lesson 1830), sea anemone ( *Anthopleura nigrescens* Verrill, 1928) (Lin & Yap submitted), snails (*Anachis misera* Sowerby 1844, *Bostrycapulus aculeatus* Gmelin 1791, *Dendropoma dragonella* Okutani & Habe, 1975, and *Nassarius* sp. Duméril 1805), chitons, serpulid polychaetes, and vent crabs (*Xenograpsus testudinatus* Ng, Huang & Ho, 2000) (Chan et al. 2016; Chen et al. 2015; Jeng et al. 2004; Wang et al. 2014; Wu et al. 2021). Within 15 m of the WV center, *A. nigrescens* (Actiniidae, Actiniaria, Cnidaria) was the dominant species, with 17.7 ± 4.5 individuals per 100 cm^2^, followed by *Bostrycapulus* snail (2.7 ± 1.1 individuals per 100 cm^2^) and *Anachis* snail (1.0 ± 0.3 individuals per 100 cm^2^) (Wu et al. 2023). Along a 50 m transect from the YV center, *Anthopleura* sea anemone covered 5% ± 4% per 25 × 25 cm^2^ (Chan et al. 2016). In WV, *Anthopleura* remained dominant at 17.5% ± 28.9% per 25 × 25 cm^2^, followed by *B. aculeatus* and *D. dragonella* snails, which ranged from 7% to 30% per 25 × 25 cm^2^.

**FIGURE 1:**
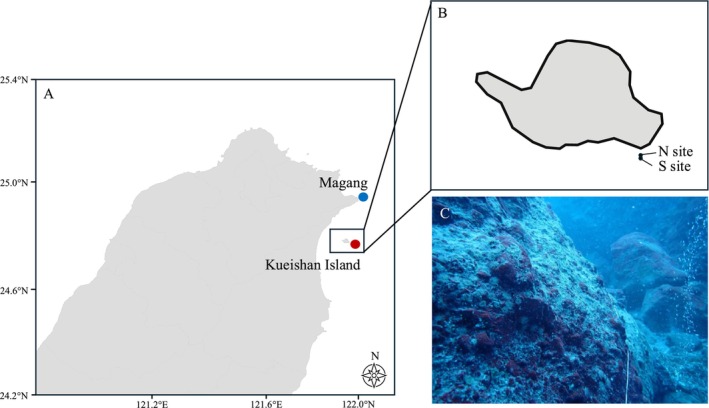
Map of the collection sites. (A) Map showing the collection sites for vent and non‐vent areas, highlighting the geographic distribution and proximity of sampling locations (A). Vent sites are marked with red dot, and non‐vent sites are marked with blue dot (B). Detailed map of the vent area, indicating the locations of the two specific sampling sites within the hydrothermal vent region (C). Photograph of samples collected on‐site at the vent area, depicting the environmental conditions and emitted fluid near the population.

Sea anemones are cosmopolitan cnidarians that thrive in a variety of marine environments. Currently, genome and transcriptome data are available for only one sea anemone species, *Alvinactis idsseensis* Zhou et al. 2023, which inhabits deep‐sea vents. These data have provided significant insights into metazoan adaptations to metal ions emitted from vent fluids, as well as to the darkness and high‐pressure conditions of the deep‐sea environment (Zhou et al. 2023). To further explore genetic adaptation to life in shallow‐water hydrothermal vent environments, we investigated the transcriptomes of *A. nigrescens* inhabiting shallow‐water hydrothermal environments in Kueishan Island. In our recent study (Lin & Yap submitted), we identified several populations near Kueishan Island, located in the northern part of Taiwan, including a non‐venting coral reef environment (Figure 1). In the present study, we conducted comparative transcriptomic analyses of *A. nigrescens* by comparing it with counterparts from the non‐venting environment and with *A. idsseensis* inhabiting a deep‐sea vent environment. Our goal was to better understand the adaptive strategies of cnidarians in extreme environments. We hypothesized that the anemones in the shallow hydrothermal vent population exhibit fewer genetic responses to hydrostatic pressure and dark adaptation while showing higher thermal tolerance compared to those in non‐venting coastal areas. The results from these comparative transcriptomic analyses provide valuable genetic insights into basal metazoan adaptability in hydrothermal vent environments and contribute to the development of genetic resources from the shallow‐water hydrothermal vent fauna.

## 2 Materials and Methods

### 2.1 Sample Collection, RNA Extraction, and Sequencing

In October 2020, *Anthopleura* specimens were collected at about 30 m away from the north and the south of the venting area (~15 m depth) off Kueishan Island (121°57′42″ E; 24°50′04″ N) by SCUBA diving. Specimens were also collected from the intertidal zone in Magang by snorkeling (122°00′06″ E; 25°00′40″ N) (Figure 1). Specimens from three populations (i.e., north of the vent, south of the vent, and Magang area) were snap‐frozen in liquid nitrogen on site and shipped with dry ice to the laboratory for RNA extraction. Whole polyps of individuals from each population (*N* = 4) were ground and subjected to RNA extraction with TRI Reagent, individually (ThermoFisher Scientific) according to the manufacturer's instructions. The quality of total RNA was examined using the Experion automated electrophoresis system (Bio‐rad). The RNA concentration and purity were assessed using Nanodrop2000 (Thermo) and Qubit Fluorometer (Invitrogen). A total of 12 cDNA libraries were constructed by the Illumina TruSeq Stranded mRNA Sample Preparation Kit and sequenced using the Illumina NovaSeq 6000 for 150 base pairs (bp) paired‐end sequencing. After sequencing, 42.6–50.2 million reads were obtained from each sample.

### 2.2 Tank Experiment

To investigate the molecular response to heat stress in vent and non‐vent anemones, a controlled tank experiment was designed. Two groups of samples, one collected from a hydrothermal vent area (Kueishan Island) and the other from a non‐vent area (Magang), were exposed to three different temperatures: 25°C, 35°C, and 45°C. Each tank was set to maintain one of these temperatures, and four individuals from each area were placed in each tank, ensuring equal representation of vent and non‐vent specimens. The anemones were acclimated in a controlled tank for 3 days to the experimental setup before being subjected to their respective temperatures for 90 min. After the exposure period, RNA was extracted immediately from three individuals in each tank to capture the transcriptional response to heat stress. cDNA libraries and RNA sequencing were performed following the method described above. Reads from each individual were mapped to the reference transcriptome using Bowtie2 (Langmead and Salzberg 2012) with default settings. The mapping results are evaluated for alignment rates and biases. Aligned reads are subsequently used to generate a count matrix that quantifies expression levels for each gene or transcript, serving as the basis for differential expression and functional analyses.

### 2.3 Reference Transcriptome Construction and Gene Annotation

A de novo transcriptome was developed incorporating samples from both vent and non‐vent areas and following the quality trimming with default settings using Trimmomatic v.0.39 (Bolger et al. 2014). Approximately 540 million trimmed reads were assembled using Trinity v2.12.0 (Grabherr et al. 2011; Haas et al. 2013). CD‐HIT v.4.8.1 was then implemented to cluster the sequences with 90% similarity (Fu et al. 2012; Li and Godzik 2006). The transcripts were translated into peptide sequences using Transdecoder v.5.5.0 (https://github.com/TransDecoder) with the default settings. The gene annotation was conducted using Blast2GO against the UniProtKB/Swiss‐prot database (accessed on June 2023) and NCBI non‐redundant protein database (accessed on September 2024) with an Evalue of 1e‐5 as the cutoff.

Single Nucleotide Polymorphism (SNPs) in samples from each location were identified using the best practices for calling variants in RNA‐seq with Genome Analysis Tool Kit (GATK) (McKenna et al. 2010). For each individual, all reads were mapped to the reference transcriptome using Bowtie2 (Langmead and Salzberg 2012), and duplicated reads were identified and removed using Picard (http://broadinstitute.github.io/picard/). Variant sites were detected using HaplotypeCaller with a minimum confidence threshold of 20, followed by genotyping using GenotypeGVCFs (McKenna et al. 2010). Variants were filtered based on FisherStrand > 30 and QualByDepth < 2 to exclude low‐quality sites. Loci that were bi‐allelic and present in at least 5% of individuals were retained using VCFtools (Danecek et al. 2011). To minimize the impact of artifacts and sequencing errors, deviations from the Hardy–Weinberg equilibrium (*q* < 0.05) were estimated and excluded from the dataset using VCFtools. Linkage disequilibrium was calculated using Plink2 (Chang et al. 2015).

### 2.4 Orthologous Search

To check the components of the assembly, orthologous proteins were searched along with the genomes and transcriptomes of anemones, *Paraphelliactis xishaensis* Feng et al. 2021 (deep‐sea nonsymbiotic) (Feng et al. 2021), and *A. idsseensis* (deep‐sea vent nonsymbiotic) (Zhou et al. 2023) and two *Anthopleura* anemones, *A. buddemeieri* (shallow‐water symbiotic) (van der Burg et al. 2016), and *A. elegantissima* (shallow‐water symbiotic) (Macrander et al. 2015) using OrthoFinder (Emms and Kelly 2019) with default settings.

### 2.5 Functional Annotation

To investigate the enrichment of Gene Ontology (GO) terms among *A. nigrescens* and anemones listed above, the orthologous groups (OGs) were identified in the following three sets: shallow_vent_set, containing the OGs present in *A. nigrescens* ; deepsea_vent_set, containing the OGs present only in *A. idsseensis*; and both_vent_set, containing OGs present in both the vent anemones. The longest contig was selected as the representative gene from each OG. For gene enrichment analysis, the R package topGO (Alexa et al. 2006) was applied to determine the significantly enriched GO terms in each dataset with a *p* < 0.05 (Fisher's exact test). To further investigate the sequence features of group‐specific transcripts/genes, homology searches were conducted using HMMER3 (Mistry et al. 2013) against a Pfam‐A database with an Evalue of 1e‐5 as the cutoff.

## 3 Results

### 3.1 Transcriptome Assembly

The de novo assembly of *A. nigrescens* consists of 64,671 contigs and 33,664 predicted protein‐coding genes, providing a substantial catalog of its functional genomic elements. The N50 value of 1576 bp indicates that the assembly's contiguity is comparable to most cnidarian transcriptomes (e.g., Kashimoto et al. 2022; Lin et al. 2017, 2019; Zhang et al. 2019). Additionally, the assembly achieved a BUSCO (Benchmarking Universal Single‐Copy Orthologs) completeness rate of 89.5%, indicating a high‐quality assembly with most expected core genes present. This high completeness rate suggests that the assembly effectively represents a significant portion of the organism's functional genome (Table 1).

**TABLE 1:** Statistics of the *Anthopleura* transcriptome assembly.

Parameter	Value
Number of raw reads pairs	276,247,369
Number of assembled bases	67,804,293
Number of contigs	64,671
N50	1576
Mean length (bp)	1048
GC content	40.61%
Number of proteins	33,664
Busco completeness	C:89.5%; F:5.8%; M:4.7%

Abbreviations: C, complete; F, frangment; M, missing.

### 3.2 Population Genetic Analysis

Variant calling and filtering identified 17,932 SNPs with a mean depth of 5.36X across 30 individuals sampled from wild conditions and tank experiments. Principal Component Analysis (PCA) (PC1: 16.4%, PC2: 14.2% of variance) and *F*st analysis (mean *F*st = 0.029) suggest that populations from Magan and the vent sites were highly connected. However, the current sample size may not be sufficient to accurately estimate allele frequencies and linkage disequilibrium, which could affect the robustness of these findings. Based on the available dataset, populations from vent and non‐vent sites appear to exhibit minimal genetic differentiation.

### 3.3 Differentially Expressed Genes Associated With Shallow‐Water Hydrothermal Vent Environment

**FIGURE 2:**
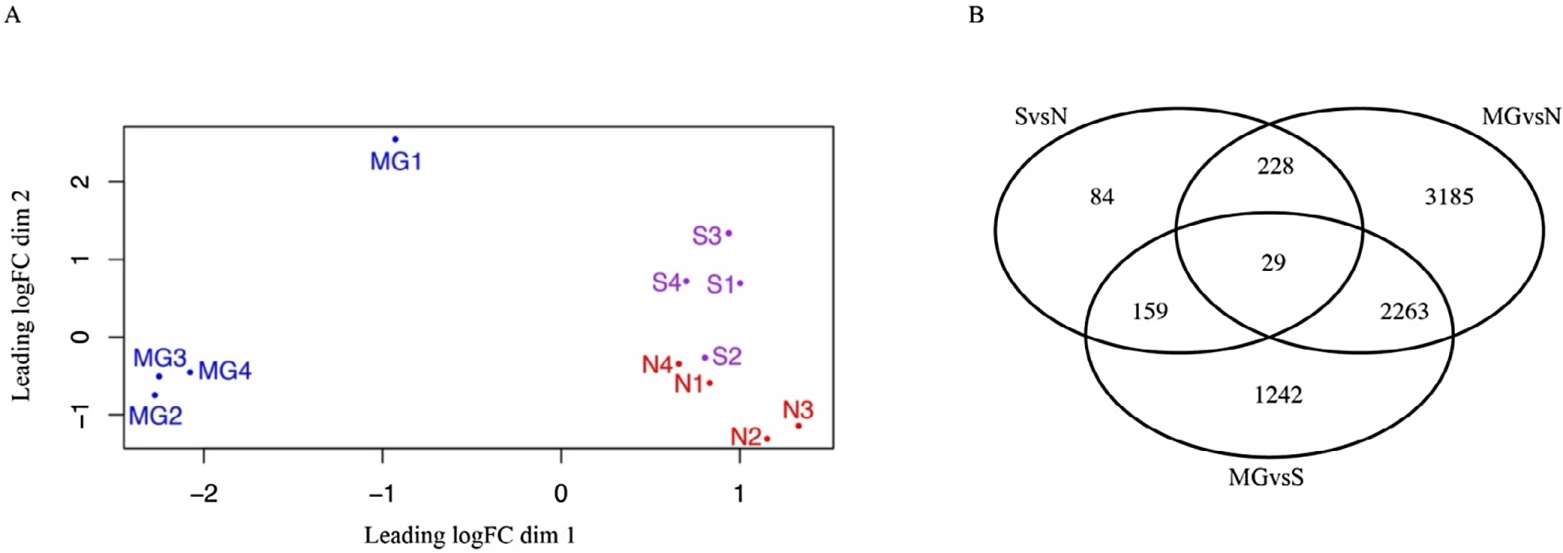
Transcriptomic comparison of three populations. (A) Multidimensional Scaling (MDS) plot illustrating the distribution of samples in this study based on gene expression profiles. Distances on the plot represent the biological coefficient of variation (BCV) between samples. BCV = 0.3924. Population in Magang is represented in blue, population at S site in purple, and population at N site in red, highlighting the clustering and divergence among the three populations. (B) The Venn diagram shows the distribution of transcripts across three comparative groups: SvsN (N site compared to S site), MGvsN (N site compared to Magang), and MGvsS (S site compared to Magang). Overlapping regions indicate DEGs shared among the groups, while non‐overlapping sections represent group‐specific DEGs.

To explore the specific response of shallow‐water hydrothermal vent environments in the sea anemone, the transcriptomic comparison was conducted across three populations: north of the vent area (N site), south of the vent area (S site), and a non‐vent area. Differential expression analysis revealed significant differences in the molecular responses of *Anthopleura* anemones to these distinct environments. The multidimensional scaling (MDS) plot revealed that samples from both vent sites—north of the chimney and south of the chimney—exhibited similar expression patterns and clustered closely together (Figure 2A). When compared to samples from the non‐vent area, more differentially expressed genes (DEGs, FDR < 0.05) were observed at the N site (> 5000 DEGs) than at the S site (> 3500 DEGs). Approximately 2300 DEGs were common to both vent populations. Specifically, 3185 DEGs were found exclusively at the N site, suggesting that the population in the north of the vent may be more strongly affected by the vent environment than those in the south (Figure 2B).

Gene ontology (GO) analysis was conducted to identify commonly enriched GOs in specimens from both vent groups. The results revealed 10 significantly enriched GOs shared by both vent populations, with similar numbers of genes involved in these GOs in samples from both sites, suggesting that both vent populations exhibit similar molecular responses. These GOs are related to molecular processes, including neural signaling regulation and transduction (GO:0090177, GO:0008227), sulfur dioxygenases (GO:0050313), and heat resistance (GO:0006468, GO:1903936). In contrast, several GOs were significantly down‐regulated, such as positive regulation of aspartate secretion (GO:1904450), glutamine family amino acid catabolic process (GO:0009065), gamma‐aminobutyric acid catabolic process (GO:0009450), positive regulation of prolactin secretion (GO:1902722), copulation (GO:0007620), glutamate metabolic process (GO:0006536), nitrogen utilization (GO:0019740), and positive regulation of heat generation (GO:0031652). To investigate the active adaptation mechanisms, the analysis focused on upregulated GOs, which are more directly relevant to survival and function in the vent environment. DEGs in both vent populations within these GOs were screened, identifying a total of 61 upregulated DEGs shared between the two vent populations (Table S1).

### 3.4 Genes Associated With H_2_S Homeostasis

Hydrogen sulfide (H_2_S) is one of the chemical compounds emitted along with the vent fluid. To investigate the anemone response to high concentrations of sulfide, the enriched GO terms and enzymes involved in H_2_S homeostasis were examined. First, genes associated with sulfur dioxygenase activity (GO:0050313) are present in the samples from both vent areas, indicating that these organisms possess the ability or plasticity to adapt to the high sulfur‐containing environment. These transcripts are primarily associated with hydroxyacylglutathione hydrolase (HAGH) and show elevated expression in the vent populations. Hydroxyacylglutathione hydrolase, also known as glyoxalase II, is a detoxification enzyme that functions to remove the toxic methylglyoxal (Kizil et al. 2000). The HAGH produces glutathione (GSH), a critical antioxidant that helps maintain redox homeostasis by neutralizing reactive oxygen species (ROS), which can become elevated due to sulfide oxidation.

Additionally, sulfurtransferases are proteins that facilitate the formation, interactions, and reactions of compounds containing sulfane sulfur atoms (Westley et al. 1983). Homologs of the sulfurtransferases family, including two evolutionarily related enzymes, thiosulfate sulfurtransferase (TST) and mercaptopyruvate sulfurtransferase (MST), have been identified in *A. nigrescens* . Three transcripts were associated with TST activity, which play a role in sulfur metabolism, iron–sulfur cluster modification, and the reduction of antioxidants such as GSH and thioredoxin (Kruithof et al. 2020). Of these, only one transcript encoding TST was significantly upregulated in vent‐dwelling populations (FDR < 0.05) (Table S1). Although not significantly upregulated, one transcript was linked with 3‐mercaptopyruvate sulfurtransferase (MPST), which is involved in H_2_S generation, tRNA thiolation, protein urmylation, and cyanide detoxification (Pedre and Dick 2021). Additionally, four transcripts encoding sulfite oxidase, an important enzyme in the sulfide‐quinone oxidoreductase (SQR) pathway, were identified, but none were significantly differentially expressed in vent populations. These findings suggest that HAGH and TST play a key role in *A. nigrescens* ' sulfur detoxification and redox homeostasis in vent environments.

### 3.5 Genes Associated With Stress Resistance

Organisms inhabiting hydrothermal vents must withstand intense thermal stress and significant temperature fluctuations (Lee et al. 2015). In cnidarians, known cellular responses to heat stress include robust mechanisms for maintaining cell membrane stability, DNA repair, oxidative stress homeostasis, and regulation of inflammatory and apoptotic pathways, as well as protein protection (Kvitt et al. 2016; Moya et al. 2016; Oakley et al. 2017; Selmoni et al. 2021). Several stress response‐associated genes previously reported in corals were found to have enriched expression in the vent populations, including the heat shock transcription factor (HSF) (Cleves et al. 2020), apoptosis regulator Bcl‐2 (Kvitt et al. 2016), melanocyte‐stimulating hormone (*MSH*) receptor (Selmoni et al. 2021)—which is also involved in circadian rhythm (see discussion)—and low‐density lipoprotein receptor‐related protein (Selmoni et al. 2021). These findings suggest that sea anemones and corals have evolved a similar genetic toolkit for responding to thermal stress, underscoring the roles of these genes in thermal stress response (Table S1). Notably, sacsin, a heat shock protein co‐chaperon proposed to be involved in coral bleaching resistance (Fuller et al. 2020), showed no differential expression in the vent anemone. This absence suggests that sacsin may represent a coral‐specific response to thermal stress (Cunning et al. 2018).

Additional significant cytoplasmic responses to stress were also observed in the vent populations. Some genes, although distributed across diverse molecular pathways, were upregulated in the vent populations. These include multiple epidermal growth factor‐like domains protein 10 (*MEGF10*), lipopolysaccharide‐binding protein, NEDD4‐binding protein 1 (*N4BP1*), and scavenger receptor cysteine‐rich type 1 protein M130, involved in the immune response; uromodulin, associated with oxidative stress; Protein C‐ets‐2 (*ETS2*), involved in development; and ETS domain‐containing protein Elk‐1 and Serine/threonine‐protein kinase mos, both of which play roles in Mitogen‐Activated Protein Kinases (MAPK) pathways.

### 3.6 G Protein‐Coupled Receptors

G protein‐coupled receptors (GPCRs) constitute a large integral transmembrane protein family with seven transmembrane domains and are responsible for the majority of cellular responses to external stimuli (Cotton and Claing 2009). Cnidarians possess the largest repertories of GPCRs among pre‐bilaterians, which are involved in neurotransmission (Krishnan and Schioth 2015), peptidergic signaling (Thiel et al. 2024), photoreception (Mason et al. 2012), and coral bleaching under heat stress (Traylor‐Knowles et al. 2017). Enriched expressed transcripts involved in G protein‐coupled receptor activity were observed in vent populations, including adenosine receptor A2a (ADORA2A), 5‐hydroxytryptamine receptor 2A, 5‐hydroxytryptamine receptor 1B, 5‐hydroxytryptamine receptor 1F, Beta‐2 adrenergic receptor, and Alpha‐1A adrenergic receptor. Among these genes, ADORA2A has the most unique genes (three unigenes and five transcripts). ADORA2A plays an important role in inhibiting inflammation through cAMP induction and attenuation of tissue damage (Ohta and Sitkovsky 2001). Serotonin (5‐hydroxytryptamine) is implicated in the stress response in stimulating the expression of heat shock protein (Tatum et al. 2015) and down‐regulating the apoptosis index of hemocytes (Jia et al. 2018). Though less is known in invertebrates, adrenergic receptors (ARs) were found to promote glycogenolysis and lipolysis to provide energy and increase survival in aquatic animals (Alexa et al. 2006; van den Thillart et al. 2002). We can speculate the GPCRs' role in mediating diverse functions, including fast repair, cell regeneration, and energy mobilization in vent anemones to recover and survive in extreme environments.

### 3.7 Genes Associated With Circadian Rhythm

Genes associated with the circadian rhythm pathway have been observed in deep‐sea hydrothermal mussels (Mat et al. 2020) and anemones (Zhou et al. 2023). In this study, two genes encoding cAMP‐responsive element modulator (*CREM*) and cyclic AMP‐responsive element‐binding protein 1 (*CREB*) were upregulated in vent populations. In mammals, the CREB/CRE transcriptional pathway is circadian‐regulated within the suprachiasmatic nuclei, which serve as the primary biological clock (Miller et al. 1996; Obrietan et al. 1999). This pathway also plays a role in the regulation of ROS detoxification (Lee et al. 2009). Though the roles of *CREM* and *CREB* in cnidarians are less understood, it was reported that the *CREB* pathway is involved in head regeneration in hydra (Kaloulis et al. 2004). Intriguingly, transcripts encoding two core genes in the invertebrate circadian rhythm pathway, *clock* and *cry2*, were upregulated in the vent populations. Although not all the genes in the circadian rhythm pathway were identified in the current transcriptome data, the upregulation of circadian rhythm‐associated genes suggests that activating these genes may be crucial for the vent anemone's survival in extreme environments.

### 3.8 Reverse Transcriptase Domain‐Containing Protein

Reverse transcriptase (RT) domains in vent organisms are essential for genetic adaptability, symbiosis, and survival in the challenging conditions of hydrothermal vent ecosystems (Lan et al. 2021; Sun et al. 2021). Among these OGs, one ortholog encoding an RT domain‐containing protein lacks a homolog in cnidarians. The closest homolog for this gene, identified against the NCBI GenBank database, is found in *Verrucomicrobiota bacterium*. This OG includes one unigene (with three transcripts) in *Anthopleura* and seven genes in *Alvinactis*, a deep‐sea hydrothermal anemone. These genes predominantly contain domains associated with reverse transcriptase (RNA‐dependent DNA polymerase) (PF0078) transposable element (TE) in both species. Reverse transcriptase activity may play a role in genome evolution (Peccoud et al. 2017). In the deep‐sea hydrothermal vestimentiferan tubeworm, putative horizontal gene transfer (HGT) genes associated with TEs of bacterial origin are believed to contribute to genome evolution (Sun et al. 2021). This finding may represent the first case of a putative HGT event in a hydrothermal vent cnidarian, warranting further investigation when the *Anthopleura* genome becomes available.

### 3.9 Orthologous Shared in Shallow‐Water and Deep‐Sea Vent Anemones

**FIGURE 3:**
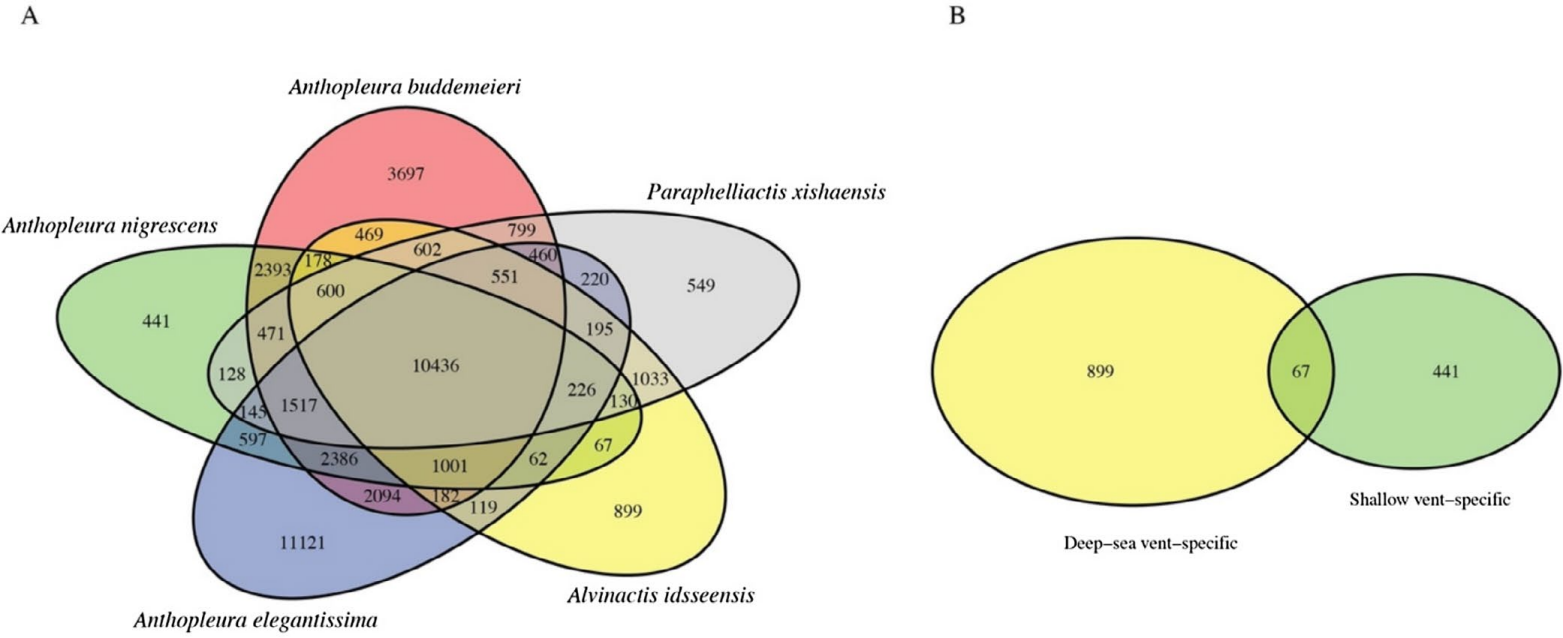
Venn diagrams of orthologous search in the sea anemones. (A) venn diagram illustrating the orthologous gene search results among five sea anemones, highlighting shared and unique genes across the selected taxa. Green indicates orthologs in A. nigrescens, blue in A. elegantissima, red in A. buddemeieri, yellow in A. idsseensis, and gray in P. xishaensis. The overlapping regions represent conserved orthologs, while non‐overlapping sections indicate species‐specific genes. (B) Venn diagram comparing orthologous genes in two groups: deep‐sea vent‐specific and shallow vent‐specific species. Sixty‐seven orthologs are shared between the two groups, while non‐overlapping areas indicate group‐specific orthologous genes associated with distinct environmental adaptations.

To further explore the common genetic machinery shared in the vent anemones, the orthologous search identified a total of 43,768 OGs in *P. xishaensis*, *A. idsseensis*, and three *Anthopleura* anemones, *A. nigrescens* , *A. buddemeieri*, and *A. elegantissima* (Figure 3A). Excluding taxon‐ or lineage‐specific OGs, the pairwise comparison revealed that 441 OGs were specific to the shallow‐water vent anemone, while 899 OGs were specific to the deep‐sea vent anemone. Only 67 OGs were shared between the two vent anemones (Figure 3B).

The GO enrichment analysis of the 67 orthologous genes revealed associations with various molecular functions. Notably, 41.8% of these genes are uncharacterized proteins or unknown genes, suggesting the possibility of novel functions in vent anemones or divergence in these genes (Table S3). The most enriched GO terms include “membrane” (genes: *SNX25*, *PCFT*, *MFSD6L*, *D5Des*, *TTN*, *ADORA3*), “ubiquitin‐protein transferase activity” (*HERC1*), transmembrane transport (*AQP3*), and “peptide‐methionine (S)‐S‐oxide reductase activity/cellular response to oxidative stress” (MSRA5). These OGs also encoded 53 pfam domains, with a substantial portion representing G protein‐coupled receptors (GPCRs), including rhodopsin‐like GPCRs, chemoreceptor Srg family (serpentine receptor class ⅴ as Srv, and serpentine receptor class ⅹ as Srx), as well as binding domains such as ANF_receptor and Peripla_BP_6, and major facilitator superfamily (MFS).

### 3.10 Differentially Expressed Orthologous Among the Vent Anemones

To elucidate the functions of the OGs shared in both shallow‐vent and deep‐vent anemones, transcriptomic analysis of the 67 genes in *A. nigrescens* populations identified six significantly differentially expressed genes (FDR < 0.05) in the populations inhabiting the vent environment. Two genes, an uncharacterized protein and a galaxin gene, were upregulated, while four genes—including an unknown gene, vitrin‐like, vegetative cell wall protein gp1‐like, and glomulin‐like—were downregulated in these populations, suggesting their essential roles in anemone evolutionary adaptation to the vent environment (Table S4).

The upregulation of a gene encoding a hypothetical protein with a XerD recombinase domain suggests its potential role in DNA repair or recombination processes (Cao et al. 1997), which are crucial in high‐stress environments like hydrothermal vents. The galaxin‐like gene, typically associated with structural proteins involved in extracellular matrix formation, plays a key role in maintaining structural integrity in marine organisms (Lin et al. 2017; Reyes‐Bermudez et al. 2009). Its upregulation in the vent population may indicate an adaptive strategy for coping with high temperatures, potentially serving as a defensive mechanism under stress conditions (Ricaurte et al. 2016; Wright et al. 2017).

Vitrin, an extracellular matrix (ECM) protein, plays a crucial role in maintaining tissue structure and organization. During injury or tissue damage, it promotes wound healing by facilitating cell migration and adhesion at injury sites (Liu et al. 2022). The downregulation of the vitrin‐like gene in the vent populations might suggest that processes requiring extensive cell adhesion or matrix remodeling are less essential in this environment, potentially due to unique ecological or metabolic demands. Vegetative cell wall protein gp1 (*GP1*), also known as hydroxyproline‐rich glycoprotein 1, is well‐studied for its role in maintaining cell wall integrity and protection in plants and algae (Ferris et al. 2001; Luo et al. 2019; Voigt et al. 2014). However, its function in animals remains largely unknown (Ryu et al. 2022; Wallberg et al. 2019). In giant freshwater prawns, *GP1* was downregulated in response to low‐temperature stress, suggesting a role in temperature stress regulation (Xing et al. 2022). The observed downregulation of *GP1* in response to environmental stress may indicate a conserved role in stress adaptation across diverse species, though its specific function in animals, particularly under extreme conditions, warrants further investigation.

The downregulation of the glomulin‐like protein, which is associated with cell differentiation and epidermal development in vertebrates (Brouillard et al. 2002; Webb et al. 2008), suggests that these processes may be deprioritized in the vent environment. However, its precise role in vent‐dwelling organisms remains unclear. Additionally, an unidentified downregulated gene, which lacks a specific match in the current GenBank database (accessed on October 2024), encodes a RecF/RecN/SMC N terminal domain involved in DNA metabolism and recombination (C. elegans Sequencing Consortium 1998). This suggests that the vent organisms may prioritize alternative molecular pathways, such as DNA repair or stress tolerance, over cell migration and tissue remodeling.

### 3.11 Gene Expression Associated With Heat Stress in Shallow‐Water Vent Anemone

**FIGURE 4:**
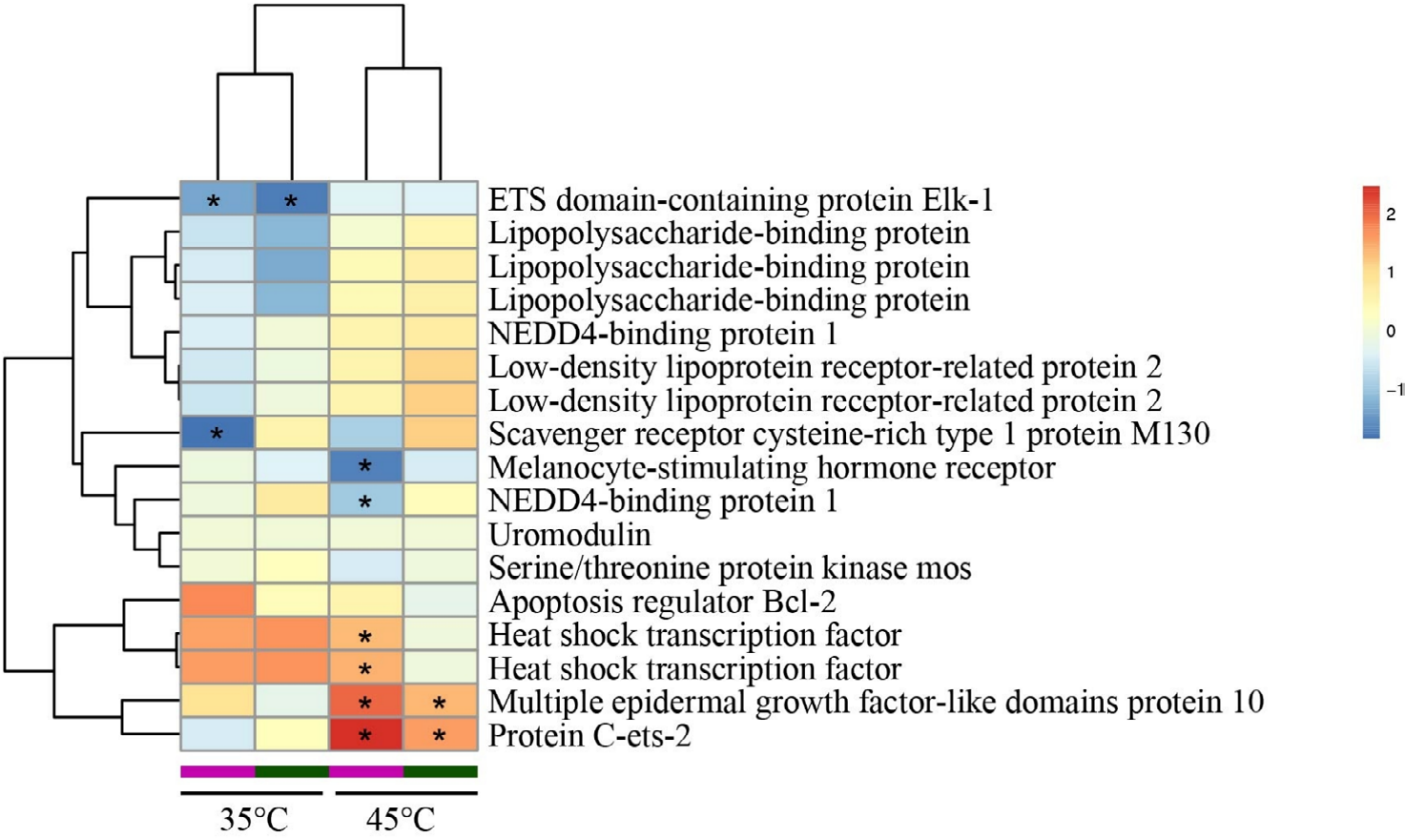
Heatmap of the expression profile of genes associated with stress resistance in response to elevated temperatures in the vent population (bars in purple) and non‐vent population (bars in green). Asterisks indicate the statistically significant value (FDR < 0.05).

Heat stress is a major challenge for vent‐dwelling sea anemones from Kueishan Island. To examine gene regulations associated with stress resistance, we conducted RNA‐seq analysis on *Anthopleura* anemones exposed to temperatures of 35°C and 45°C. The results showed that the vent population exhibited greater activation of specific biological processes in response to elevated temperatures, particularly at 45°C, compared to the non‐vent population. This suggests that the hydrothermal vent population has developed unique molecular regulatory mechanisms for heat adaptation. In response to heat stress, vent anemones displayed significant enrichment of differentially expressed genes, including the up‐regulation of *HSF*, *MEGF10*, and *ETS2*, while genes such as the *MSH* receptor, ETS domain‐containing protein Elk‐1, and *N4BP1* were downregulated. Notably, the ETS domain‐containing protein Elk‐1, *MEGF10*, and *ETS2* were also differentially expressed in non‐vent individuals, following similar expression trends. This highlights a conserved stress response mechanism shared among *Anthopleura* anemones (Figure 4).

## 4 Discussion

*A. nigrescens* exhibits remarkable ecological ability to thrive across a wide range of environments, from shallow‐water hydrothermal vents to dynamic rocky intertidal zones. In the vent ecosystem, this species is particularly dominant, likely due to its tolerance to elevated temperatures and hydrogen sulfide exposure. In contrast, in rocky intertidal habitats, *A. nigrescens* coexists with other sea anemones such as *Actinia* spp., where fluctuating environmental stressors, including desiccation and wave action, influence its distribution and abundance. This broad environmental plasticity suggests that *A. nigrescens* possesses unique physiological and biochemical mechanisms that enable survival under varying selective pressures. Using the transcriptomics approach, this study contributes to understanding these adaptive mechanisms in hydrothermal vent environments, paving the way for future research on how these traits may also facilitate its persistence in other extreme habitats, such as the highly variable intertidal zone.

The high‐quality de novo transcriptome assembly of *A. nigrescens* provides a robust foundation for exploring its functional genomics, particularly in relation to environmental stress adaptations. Differential expression analysis reveals significant variations across populations from vent and non‐vent areas. Most of the enriched GOs identified reflect the molecular response of *Anthopleura* to the unique vent environment, especially those involved in heat resistance and detoxification mechanisms. Samples from vent populations, particularly those north and south of the chimney, exhibited clustering patterns indicative of similar transcriptional responses to the vent environment, with notable enrichment in genes related to sulfur metabolism and heat resistance.

The northern population exhibited a greater number of unique DEGs, suggesting that the direction of hydrothermal vent emissions may intensify environmental pressures, leading to more distinct molecular responses. Studies on the diffusion activities of hydrothermal vents near Kueishan Island indicate interactions between the Kuroshio Current and tidal currents (Han et al. 2014; Hsu et al. 2021). The Kuroshio Current primarily flows from the south, causing the northern part of the vent to experience stronger influence from the vent discharge. Consistent with this diffusion pattern, a study on the shell traits of dove snails inhabiting this vent area found that snails in the northwest section exhibited reduced shell thickness compared to those in the southern region. This suggests that varying degrees of exposure to hydrothermal plume, influenced by water movement, impact the ecophysiological performance of shelled organisms (Chen et al. 2014). These findings imply that benthic organisms, such as *Anthopleura* anemones, in the northern region are more significantly affected by hydrothermal vent diffusion than those in the south, where tidal forces and the predominant northward movement of the Kuroshio Current play a strong role.

### 4.1 Detoxification Adaptation

The distribution range of the elemental sulfur and hydrogen sulfide emitted originating from the shallow‐water hydrothermal vent off Kueishan Island is approximately 0.5 km^2^ (Chen, Zeng, et al. 2005). These emissions influence the marine ecosystem, impacting factors such as the distribution pattern and sexual dimorphism of crabs (Tseng et al. 2020) and the composition of fish larvae assemblages (Hsieh and Lo 2019). Although H_2_S is toxic to many organisms, studies have identified genes associated with H_2_S detoxification in aquatic animals (Kelley et al. 2016; Tobler et al. 2018). In this study, genes linked to H_2_S homeostasis in *A. nigrescens* exhibited differential expression between vent and non‐vent populations. The significant upregulation of HAGH and TST transcripts in vent populations suggests that these enzymes play key roles in managing sulfur toxicity and maintaining redox balance. Notably, research on the last universal common ancestor (LUCA) suggests that detoxification enzymes such as HAGH and TST are ancient and likely evolved in response to early Earth's extreme environments, analogous to hydrothermal vents (Ouzounis et al. 2006). However, without direct biochemical or physiological validation, the precise function of these genes in H_2_S detoxification remains uncertain. It is possible that these enzymes contribute to the detoxification process, but further investigation is needed to confirm their roles in *A. nigrescens* ' adaptation.

In the deep‐sea hydrothermal vent sea anemone *Alvinactis* sp., the oxidation of sulfide into less toxic forms, such as thiosulfate, has been linked to vent‐specific genes encoding sulfur dioxygenase, as well as differentially expressed genes involved in oxidative phosphorylation (Xu et al. 2022). In contrast, sulfite oxidase, a key enzyme in the sulfide‐quinone oxidoreductase (SQR) pathway, was not significantly upregulated in *A. nigrescens* vent populations. This suggests that *A. nigrescens* may rely more on HAGH and TST enzymes for sulfur detoxification and redox balance, though alternative pathways cannot be ruled out. The vent crab inhabiting Kuishan Island appears to primarily rely on sulfide oxidation to thiosulfate as its major detoxification mechanism (Chang 2007). Increased copy numbers of sulfite oxidase and thiol sulfurtransferase associated with the SQR pathway have been observed in the genome of *A*. *idsseensis* (Zhou et al. 2023). If gene expansion of sulfite oxidase is a common adaptation among vent anemones, *A. nigrescens* may maintain baseline expression of this enzyme sufficient to handle ambient sulfide concentrations while relying on other pathways for additional sulfur detoxification.

The function of the RT‐containing protein in *A. nigrescens* remains unclear, but its sequence similarity to *Verrucomicrobiota* suggests a potential role in degrading sulfated methyl pentoses (Orellana et al. 2022). In hydrothermal vent systems, annelids produce mucus rich in methyl monosaccharides, which may serve a protective function (Talmont and Fournet 1990). Sulfur isotopic analyses indicate a chemoautotrophic source of sulfur, derived from dissolved sulfide in vent fluids in vent animals, rather than sulfur originating from seawater sulfate through photosynthesis (Erickson et al. 2009). Additionally, endosymbionts contribute to H_2_S detoxification and energy metabolism in hydrothermal vent mussels (Sun et al. 2022) and vent crabs (Chou et al. 2023). The cycling of organic sulfur compounds in hydrothermal systems is likely driven by biotic processes and may be prevalent within hydrothermal vent microbial communities (Rogers and Schulte 2012). However, *A. nigrescens* appears to rely primarily on plankton‐derived food sources, such as dead zooplankton killed by sulfurous plumes (Jeng et al. 2004; Wu et al. 2021), indicating a predominantly nonsymbiotic lifestyle. While the RT‐containing protein may contribute to methyl pentose degradation activity and/or sulfur metabolism in *A. nigrescens* , further studies are required to determine its specific function. Similarly, the potential interactions between symbiotic microbes and vent anemones warrant deeper investigation.

### 4.2 Stress Resistance Adaptation

The upregulation of stress‐response genes highlights the resilience of hydrothermal vent‐dwelling anemones and provides insights into both shared and distinct molecular responses of cnidarians to extreme environments. Studies on basal metazoan apoptotic responses indicate that corals possess apoptotic networks similar to those found in higher animals (Kvitt et al. 2016). In the deep‐vent anemones, gene family expansion related to the stress response, including DNA repair and immune response, has been observed (Zhou et al. 2023). In *Anthopleura* anemones, genes associated with the thermal stress response exhibit patterns similar to those in corals and deep‐vent anemones, suggesting a conserved genetic toolkit for survival in extreme environments. Key genes identified in the vent population, such as the *HSF*, apoptosis regulator Bcl‐2, melanocyte‐stimulating hormone receptor, XerD recombinase domain‐containing protein, and a galaxin‐like gene, play roles in maintaining cellular integrity, regulating oxidative stress, and supporting DNA repair. This shared genetic toolkit suggests that thermal stress adaptations in sea anemones and corals may have evolved convergently or have been retained from a common cnidarian ancestor, reflecting shared survival strategies in fluctuating and extreme temperature conditions. Additionally, efficient DNA repair appears to be a crucial component of the stress resistance response to thermal stress. Notably, sacsin, a heat shock protein co‐chaperone linked to coral bleaching resistance, showed no differential expression in vent anemones, supporting a coral‐specific adaptation (Cunning et al. 2018). This divergence indicates that while fundamental stress‐response mechanisms are conserved, species‐specific pathways evolve to address unique environmental challenges.

The identification and upregulation of circadian rhythm‐associated genes in vent populations of *A. nigrescens* suggest that these molecular pathways may help the anemones respond to environmental fluctuations in hydrothermal vents. The CREB/CRE transcriptional pathway, which is regulated by the circadian clock in the suprachiasmatic nuclei of animals, plays a key role in maintaining biological rhythms (Miller et al. 1996; Obrietan et al. 1999). Similarly, the upregulation of *clock* and *cry2*, two core genes in the invertebrate circadian rhythm pathway, suggests that these genes may contribute to the physiological responses of vent anemones in highly dynamic hydrothermal vents. However, without behavioral data or diel gene expression profiling, the functional significance of these genes in vent adaptation remains uncertain. Hydrothermal vent activity off Kueishan Island is influenced by tidal currents and the Kuroshio Current (Chen, Zeng, et al. 2005; Hsu et al. 2021), leading to fluctuations in environmental conditions such as temperature and nutrient availability. Since vent activity can impact food availability (Jeng et al. 2004), further research is needed to determine whether circadian rhythms play a role in optimizing feeding efficiency and survival in *A. nigrescens* .

### 4.3 Molecular Targets for Heat Stress in Shallow‐Water Vent Anemone

By employing RNA‐seq analysis to evaluate gene expression responses to elevated temperatures, the results reveal distinct patterns of molecular adaptation between the hydrothermal vent and non‐vent populations under heat stress, particularly at 45°C, highlighting the adaptive strategies evolved by the vent population. This observation suggests that the hydrothermal vent population has developed unique molecular regulatory mechanisms to cope with extreme and fluctuating thermal conditions. Similar to other animals, HSFs play a crucial role in regulating genes in response to thermal stress (Cleves et al. 2020; McMillan et al. 1998). In coral, *MEGF10*, which is involved in phagosome formation, is upregulated to facilitate the removal of invading pathogens and/or the clearance of damaged apoptotic cells (Libro et al. 2013). The clearance of damaged apoptotic cells is likely a critical thermal adaptation in vent sea anemones. ETS2 and ETS1, evolutionarily conserved across the metazoans (Degnan et al. 1993), are required for endothelial cell survival in mammals (Wei et al. 2009) and play roles in anti‐infection processes in invertebrates (Ma et al. 2009). Compared to the closely related ETS1, less is known about the regulatory mechanism in ETS2, particularly in basal metazoans, though evidence suggests that these genes have overlapping roles in development and immunity (Garrett‐Sinha 2013; Wei et al. 2009).

Notably, the differential expression of the *MSH* receptor in vent individuals may reflect a specialized stress‐response mechanism and energy homeostasis under elevated temperature. Cutaneous melanin plays an important role in daily circadian variation in invertebrates (Bertolesi et al. 2016). *MSH* influences the expression of clock genes involved in rhythmic processes in vertebrates (Moraes et al. 2014) and has also been linked to heat stress in corals (Selmoni et al. 2021). Additionally, an increased copy number of *MSH* receptors has been observed in deep hydrothermal vent sea anemones (Zhou et al. 2023), suggesting that the *MSH* factor is crucial for maintaining rhythmic cycles and thermal adaptation in these organisms. Further investigation into the regulatory mechanisms of the *MSH* factor is warranted. The specialization of these genes may reflect localized selection pressures, enabling the hydrothermal vent population to thrive in such challenging environments. This underscores the unique adaptive strategies employed by hydrothermal vent invertebrates for thermal resilience and survival in extreme conditions (Zhang et al. 2024).

Some genes are differentially expressed in similar trends in both vent and non‐vent populations. These findings support the hypothesis that thermal stress elicits both shared and population‐specific genetic responses in *Anthopleura* anemones. Conserved response mechanisms likely provide a foundational adaptation to thermal fluctuations, while the unique regulatory pathways observed in the vent population demonstrate evolutionary plasticity in response to extreme habitats. Further studies focusing on the functional roles of these genes, combined with analyses of broader environmental factors, could offer deeper insights into the molecular basis of stress resistance and the evolutionary dynamics of these anemones.

## 5 Conclusion

This study sheds light on the molecular adaptations of *A. nigrescens* to extreme shallow‐water hydrothermal vent environments. By integrating RNA‐seq analyses, comparative genomics, and functional predictions, we uncovered both shared and population‐specific genetic responses that underpin the resilience of vent and non‐vent populations to thermal and chemical stressors. The conserved expression of stress‐response genes, such as those involved in H_2_S detoxification and thermal regulation, highlights foundational mechanisms that enable *Anthopleura* anemones to survive environmental fluctuations. The results also indicate GPCRs' role in mediating diverse functions, including fast repair, cell regeneration, and energy mobilization in vent anemones to recover and survive in extreme environments. Meanwhile, the unique upregulation of key genes in vent populations, including *HAGH*, *TST*, circadian rhythm regulators, and *MSH* receptor genes, demonstrates evolutionary plasticity tailored to the challenges of hydrothermal vent habitats.

Our findings emphasize the importance of detoxification, redox balance, DNA repair, and circadian regulation as critical strategies for survival in extreme conditions. Notably, the specialized roles of *MSH* receptors in maintaining energy homeostasis and rhythmic cycles suggest adaptive significance in coping with elevated temperatures and dynamic vent conditions. Furthermore, the differential gene expression trends shared between vent and non‐vent populations underscore the interplay between common cnidarian survival strategies and localized selection pressures. These results offer significant insights into the genetic and molecular basis of stress resistance in vent‐dwelling sea anemones and their evolutionary trajectories. Future research focusing on the functional characterization of key genes, coupled with broader ecological and environmental context analysis, could deepen our understanding of how these organisms thrive in such hostile environments. Additionally, although the interaction between the *Anthopleura* and its associated microbiomes is understudied, specific microorganisms, such as sulfur‐oxidizing bacteria and heat‐resistant symbionts, which participate in the biological processes of *A. nigrescens* , particularly in thermal resistance and H_2_S detoxification, warrant further investigation. This study provided the first transcriptomic analyses from the basal metazoans inhabiting shallow‐water hydrothermal vents, shedding light on the evolution of the genetic toolkits responding to the extreme environment.

## Author Contributions

**Mei‐Fang Lin:** conceptualization (equal), data curation (lead), formal analysis (lead), funding acquisition (equal), resources (equal), software (lead), writing – original draft (lead), writing – review and editing (equal). **Li‐Lian Liu:** conceptualization (equal), funding acquisition (equal), project administration (equal), resources (equal), writing – original draft (equal). **Chen‐Tung Arthur Chen:** conceptualization (equal), funding acquisition (equal), project administration (equal), resources (equal).

## Conflicts of Interest

The authors declare no conflicts of interest.

## Supporting information


Tables S1–S4.

## Data Availability

The transcriptomic raw data used in this study have been deposited in SRA‐NCBI under the BioProject accession number PRJNA1042849.
